# The Role of Serial Liquid Biopsy in the Management of Metastatic Non-Small Cell Lung Cancer (NSCLC)

**DOI:** 10.3390/clinpract12030046

**Published:** 2022-06-10

**Authors:** Srikar Sama, Thuy Le, Asad Ullah, Islam A. Elhelf, Sravan K. Kavuri, Nagla Abdel Karim

**Affiliations:** Georgia Cancer Center, Medical College of Georgia Augusta University, Augusta, GA 30912, USA; srikar.sama@gmail.com (S.S.); tle5@augusta.edu (T.L.); aullah@augusta.edu (A.U.); ielhelf@augusta.edu (I.A.E.); skavuri@augusta.edu (S.K.K.)

**Keywords:** non-small-cell lung cancer (NSCLC), sotorasib, liquid biopsy, mutation

## Abstract

Lung cancer is the leading cause of cancer-related deaths. Surgery remains the best option to treat lung cancer when feasible. However, many cases are diagnosed beyond the initial stages. There has been tremendous progress in the treatment of lung cancer over the last few years. Studies have shown that biomarker-driven targeted therapies lead to better outcomes. Due to the technical difficulties and significant procedural risk associated with repeated tissue biopsies, analysis of tumor constituents circulating in the blood, such as circulating tumor DNA (ctDNA) and various proteins, is becoming more widely recognized as an alternative method of tumor sampling, i.e., liquid biopsy. Liquid biopsy is superior to tissue biopsy, as it is minimally invasive and easily repeatable. Given the recent data on changes in mutations as the disease progresses or responds to treatment, liquid biopsies can help monitor the changes and guide us in giving targeted drugs. Here we present a case of advanced NSCLC who was initially started on Alectinib based on positivity for *ALK* gene rearrangement found in the FISH study. At the time of progression, molecular profiling liquid biopsy was obtained, which revealed *KRAS*-*p.G12C* mutation. Thus, the patient’s therapy was later on changed to sotorasib after the FDA approved a *KRAS-p.G12C* mutation inhibitor.

## 1. Introduction

Lung cancer is classified into small cell lung cancer (SCLC) and non-SCLC (NSCLC); NSCLC accounts for approximately 85% of all lung cancer cases [1]. Lung cancer remains the number one killer among cancers worldwide [2]. Smoking continues to be the most important risk factor for lung cancer, including secondhand smoke [3]. However, aggressive smoking cessation programs are not enough to prevent lung cancer. Biomarker-driven targeted therapies are required to improve clinical outcomes for patients meaningfully. Most NSCLC patients undergo systemic therapy, either being diagnosed at an already inoperable stage or experiencing disease relapse after surgery [4]. Next-generation sequencing (NGS) is being used to test for tumor mutations. The clinical applications of NGS will further increase as technology, bioinformatics, and resources improve to address the limitations and improve the quality of results.

The detection of *EGFR*, *BRAF*, and *MET* mutations and the analysis of *ALK*, *ROS1*, *RET,* and *NTRK* translocations have already been incorporated into NSCLC diagnostic guidelines, and their inhibitors are recommended whenever indicated. NSCLCs are also subjected to the analysis of PD-L1 protein expression in order to direct the use of immune checkpoint inhibitors.

## 2. Case Presentation

A 71-year-old gentleman was referred to the Oncology clinic for evaluation of a 2.5 cm left upper lobe lung nodule with a large left-sided pleural effusion observed at the academic hospital emergency department. Urgent thoracentesis was not performed, as the patient had no significant symptoms.

He had been in his usual state of health until the first week of July 2020, when he started to experience lower left-sided back pain, radiating to the left side and eventually radiating to the epigastric. The patient underwent extensive workup with gastroenterology, including esophagogastroduodenoscopy (EGD) and magnetic resonance imaging (MRI) abdomen, which were negative.

**Figure 1:**
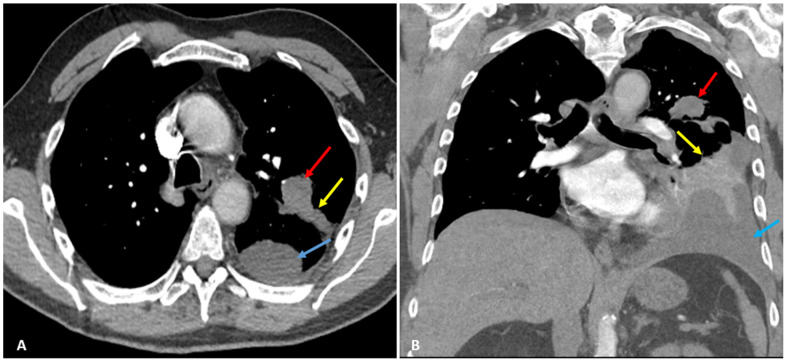
Axial (**A**) and coronal (**B**) CT scan with IV contrast shows left upper lobe peri-bronchial mass (red arrows) measuring approximately 3 × 2 cm in diameter. Distal obstructive atelectasis (yellow arrows) and left pleural effusion (blue arrows) are observed as well.

Later, he started to experience excruciating left lateral chest pain, radiating to his shoulder and neck, 10/10 in intensity, and worsened with deep inspiration, leading to shallow breathing and dyspnea. He returned to his primary physician, who ordered a chest X-ray (CXR) and computed tomography (CT) chest with intravenous (IV) contrast revealing left upper lobe peri-bronchial mass (red arrows) measuring 3 × 2 cm in diameter, distal obstructive atelectasis, and left pleural effusion (Figure 1).

**Figure 2:**
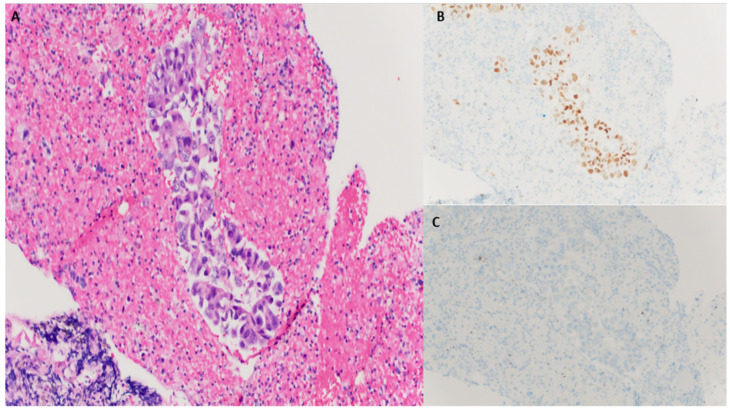
(**A**), H&E 20×, tumor cells forming incomplete lumina with hyperchromatic nuclei; (**B**) Strong nuclear staining of tumor cells with TTF1; (**C**) Tumor cells are negatively stained for P40.

He was then sent to the emergency department to seek immediate medical attention. The patient reported a 13 lbs. (6 kg) intentional weight loss over the period of 2 months due to drastic changes in his diet in an effort to lose weight. A biopsy of the pulmonary nodule was performed. The histologic examination revealed irregular glandular clusters with hyperchromatic nuclei, nuclear pleomorphism, and abundant eosinophilic vacuolar cytoplasm. The tumor cells were strongly positive for transcription termination factor 1 (TTF1) and negative for P40. Based on morphology and immunohistochemical patterns, a diagnosis of adenocarcinoma was rendered (Figure 2). Genomic profiling and study timeline are described (Table 1).

Fluorescence in situ hybridization (FISH) study by using Vysis ALK break-apart probe showed 16% of cells were positive for an *ALK* rearrangement. PET scan showed a left upper lobe posterior apical lung adenocarcinoma mass 3.1 × 2.2 cm (stage T2a). The subcarinal left para-aortic and left hilar region were positive for metastatic disease (stage N2). Multiple left lung pleural metastases were also seen (stage M1a). He was diagnosed with clinical Stage IVA lung adenocarcinoma. Brain MRI was negative for extra or intracranial metastasis. He was started on Alectinib 600 mg PO BID (an ALK TKI) based on *ALK* rearrangement. The molecular profiling of the tumor cells revealed wild-type *EGFR, KRAS/NRAS*, and *ROS1* rearrangement.

Interim PET scan after 2 months of ALK inhibitor therapy showed progression of the disease and new osseous bone metastases. Therapy was changed to Bevacizumab/Atezolizumab/Carboplatin/Paclitaxel s/p 4 cycles. A follow-up PET scan at that time showed a >20% increase and, thus, disease progression per review in the thoracic tumor board. At that time, liquid biopsy for molecular profiling revealed *KRAS-p.G12C* mutation and no *ALK* rearrangement. Liquid biopsy was obtained, and cell-free DNA was isolated from whole blood. Following DNA library preparation, next-generation sequencing (NGS) of specific gene regions was performed. In this particular case, we used a commercially available assay called guardant 360 for genomic profiling. The test detects single nucleotide variants in a targeted panel of 83 genes and selected copy number amplifications, fusions/rearrangements, and indels for a specific set of genes [5]. Yet, at that time, the therapy for such mutation was not available given the lack of FDA approval. On further disease progression, the patient was subsequently started on sotorasib in July 2021 after the FDA granted approval for *KRAS-p.G12C* targeted agent. The patient achieved a mixed response noted on restaging scans in September 2021. However, he continued to progress while on sotorasib and was eventually enrolled in hospice care after 4 months of sotorasib.

## 3. Discussion

Lung cancer is a molecularly heterogeneous disease, and insight into its biology is essential for developing effective therapies. The treatment of lung cancer has changed from the empirical use of chemotherapy to mutation-targeted therapies.

The rise of the personalized era in lung cancer prompted the evaluation of novel diagnostic tools to overcome some of the limitations of traditional tumor genotyping. The ability to obtain adequate tissue from the lung or metastatic sites may be limited due to the patient’s performance status or the risks associated with the procedures.

Liquid tumor biopsies have sparked a great deal of interest in the oncology community [6]. Liquid biopsy refers to a multitude of minimally invasive techniques that can allow real-time biomolecular characterization of the tumor through the analysis of human body fluids [7]. These somatic alterations can be determined using a variety of biomarkers, the most well studied and widely used of which are tests that analyze circulating tumor DNA (ctDNA).

While ctDNA analysis by liquid biopsy appears to be most well defined for the EGFR T790M mutation, they seem to be equally valid for other driver mutations such as ALK, ROS1, and NTRK, and the detection of resistance mutations for these driver mutations [8,9]. Analysis of ctDNA has been shown effective in detecting evidence of the T790M mutation with comparable accuracy to that of traditional tissue biopsy [10]. Liquid biopsy has also revealed *KIF5B-RET* fusions in patients who had previously tested negative for *KIF5B-RET* fusions in tissue samples [11].

The significance of liquid biopsy in identifying new mutations can determine a change of treatment. In a case report by Suppiah et al. [12], a patient’s therapy was changed to afatinib after an *EGFR* exon 19 deletion was identified by liquid biopsy, which was missed on a tissue biopsy. In another case report by Dietz et al. [13], rising allelic frequencies of the *ALK* fusion were detected by liquid biopsy, which led to a change in chemotherapy from crizotinib to ceritinib. Analysis of ctDNA for molecular characterization of acquired resistance was also shown in a case series by Bordi et al., in which *ALK* point mutations were identified in 5 of 20 NSCLC patients treated with crizotinib who showed disease progression. Following that, Bordi and the team reported that *ALK* and *KRAS* mutations are linked with acquired crizotinib resistance in *ALK*-positive NSCLC [14]. Thus, treatment decision-making is becoming even more individualized owing to liquid biopsy.

In the case presented here, the patient’s disease has been difficult to control with the current standard of care. He progressed after the 2nd line of therapy using platinum-based chemotherapy and taxane-based chemotherapy with or without antiangiogenic therapy. Pemetrexed was also utilized as the chemotherapy backbone on the treatment arm via clinical trial after he had progressed on 2nd line therapy. Then a change of therapy to sotorasib was promptly initiated after the *KRASp.G12C* mutation inhibitor was approved by the FDA in late May 2021.

The *KRAS* gene is the most frequently mutated oncogene in human cancers. It encodes a guanosine triphosphatase (GTPase) that cycles between active guanosine triphosphate (GTP)-bound and inactive guanosine diphosphate (GDP)-bound states to regulate signal transduction [15,16]. The *KRAS p.G12C* mutation occurs in approximately 13% of non-small cell lung cancers (NSCLCs) [17]. The glycine-to-cysteine mutation at position 12 favors the active form of the KRAS protein, resulting in a predominantly GTP-bound KRAS oncoprotein and enhanced proliferation and survival in tumor cells [18].

Sotorasib showed anticancer activity in patients with *KRAS p.G12C*-mutated advanced solid tumors in a phase 1 study, and particularly promising anticancer activity was observed in a subgroup of patients with non-small cell lung cancer (NSCLC) [19]. Sotorasib also showed clinical efficacy with reversible toxic effects, mainly of grade 1 or 2, in the phase 1 portion of the CodeBreaK100 trial [20].

In the NSCLC cohort of a phase 2 portion trial, an objective response was observed in 37.1% of patients, with a median duration of response of 11.1 months. The median progression-free survival was 6.8 months, and the median overall survival was 12.5 months. In addition, tumor shrinkage and disease control were observed in the majority of patients. These data provide further evidence in support of the clinical use of sotorasib in patients with *KRAS p.G12C*-mutated NSCLC [19].

For the future perspective, studies have shown that serial liquid biopsies of *KRAS* mutant NSCLC are correlated with clinical outcomes. The early assessment of NSCLC has the potential for monitoring outcomes in patients with NSCLC [21]. The study by Heitzer et al. evaluated the role of liquid biopsy in NSCLC. The results of their study suggested that liquid biopsy is helpful in cases when resistance to management is suspected, patients with discordant clinical history, and tumors with heterogeneity (intertumoral and intratumoral). They also suggested that liquid biopsy can help in situations when tumor locations are hard to biopsy and there is insufficient sampling on cytology/biopsy [22].

ALK fusion NSCLC is associated with heterogeneous clinical outcomes. A study by Wang et al. demonstrated the prognostic value of *EML4-ALK* fusion variants with the clinical outcomes in patients. The results of their study showed patients with variant 1 for *EML4-ALK* fusion are associated with equivalent overall survival (OS) and progression-free survival (PFS) with non-v1 variant patients. Patients with v3 and non-v3 had similar PFS. However, v3 had worse OS than non-v3 patients [23]. *ALK/KRAS* comutations are associated with resistance to ALK TKI. The outcomes with ALK and EGFR TKI are inferior in patients with either mutation alone [24]. Noordhof et al. demonstrated that *KRAS* mutation has no prognostic significance in treating patients with pembrolizumab when PD-L1 expression is >50% in stage IV lung adenocarcinoma. The survival was similar in patients with *KRAS* mutated versus *KRAS* wild-type in NSCLC when PD-L1 expression was >50% when pembrolizumab was used as first-line therapy. In selected patients with PD-L1 > 50%, *KRAS* mutations were more frequent in women in comparison to men [25].

## 4. Conclusions

The routine use of established liquid tumor biopsies in the management of non-small cell lung cancer should be considered in any case when the available ‘solid’ tissue does not allow for the important evaluation of the presence of a clinically validated ‘actionable’ molecular target. Our case study demonstrates the potential clinical utility of liquid biopsy for analyzing mechanisms of treatment failure and predicting future clinical outcomes and also mentions the newly approved drug sotorasib for *KRAS p.G12C* mutation.

## Figures and Tables

**Table 1:** Oncology history and treatment timeline.

Pathologic Diagnosis and Molecular Profiling	Oncology Treatment	Treatment Duration
Tissue biopsy in August 2020 with ALK rearrangement	Alectinib	September 2020–November 2020
Liquid biopsy in November 2020 with *KRAS p.G12C* mutation	Bevacizumab/atezolizumab/carboplatin/paclitaxel	November 2020–January 2021
	Bosutinib/pemetrexed via Phase 1 clinical trial	January 2021–July 2021
	Sotorasib after FDA approval	July 2021–November 2021

## Data Availability

No new data were created or analyzed in this study. Data sharing does not apply to this article.
